# Computer Assisted Objective Assessment of Micro-Neurosurgical Skills From Intraoperative Videos

**DOI:** 10.1109/OJEMB.2023.3257987

**Published:** 2023-03-16

**Authors:** Pon Deepika, Kalahasti V. V. Deepesh, Phani Sriram Vadali, Madhav Rao, Vikas Vazhayil, Alok Mohan Uppar

**Affiliations:** IIIT-Bangalore 29129 Bangalore 560100 India; Department of NeurosurgeryNIMHANS 29148 Bangalore 560029 India

**Keywords:** Computer vision, mask RCNN, micro-neurosurgery, motion analysis, skill assessment, Yolov5

## Abstract

*Goal:* Conventionally, a surgeon's skill is assessed through visual observation by experts and by tracking patient outcomes. These techniques are very subjective and demands enormous time and effort. Hence, the aim of this study is to construct a framework for automated objective assessment of micro-neurosurgical skill. *Methods:* A mask region-based convolution neural network (RCNN) is trained to identify and localize instances of surgical instruments from the recorded neurosurgery videos. Then the tool motion and tool handling metrics are computed by tracking the detected instrument locations through time. Microscope adjustment patterns are also investigated via the proposed time based metrics.*Results:* This study highlights the metrics that could potentially emphasize the variance in expertise between a veteran and a novice. These variations include an expert exhibiting a lower velocity, lower acceleration, lower jerks, reduced path length, higher normalized angular displacement, increased bi-manual handling, shorter idle time and smaller inter tool-tip distances while handling tools accompanied with frequent microscope adjustments and reduced maximum and median intervals between adjustments when compared to a novice. *Conclusions:* The developed vision based framework has proven to be a reliable method to assess the degree of surgical skill objectively and offer prompt and precise feedback to the neurosurgeons.

## I. Introduction

Central Nervous System is incredibly complicated with various interconnections that involve all other major organs and glands [1]. This makes neurosurgery as one of the highly demanding, time-consuming and the most challenging field with huge consequences for the committed errors [2]. Neurosurgery necessities a broad range of precise and highly complex technical skills from the residents. An analysis of 3063 Colorectal and noncolorectal procedures performed by 17 surgeons from 2014 to 2016 in a quality improvement study by Illinois Surgical Quality Improvement Collaborative (ISQIC) highlights that there is a strong positive association between technical proficiency of a surgeon and patient outcomes in terms of unplanned hospital re-admission, serious infections, unplanned re-operation in relation to the primary procedure, mortality or serious morbidity [3]. To achieve competency in neurosurgical skills and to manipulate surgical tools under the operating microscope require extended periods of training and continuous feedback.

Checklist based surgical skill assessments like Structured Assessment of Technical Skills (OSATS), Operative Performance Rating System (OPRS), Multiple Objective Measures of Skill (MOMS) are prone to evaluator bias and offer limited feedback to the trainee residents besides demanding huge time and effort from the experts [4]. The existing challenges fueled by the recent advances in computational ability and machine learning techniques paved way for a new paradigm of automated system for assessment and feedback of surgical competencies which is more precise and repeatable. In this regard, objective measures of complex psychomotor skills of neurosurgeons that are reproducible are introduced in the paper. The operating microscopes offer greater visibility in the regions around deep cavities or lesions and forms an integral tool for neurosurgical activities. The knowledge of neurosurgical maneuvers are not practical unless combined with appropriate adjustments in settings and positioning of the operative microscope. Hence, microscopic adjustments and its associated features characterizes a skill component of the operator which is also included in this article.

The objective of this study is

1) To automate the assessment of micro-neurosurgical skills in the recorded neurosurgery videos through the introduction of metrics to apprehend the surgeons' tool and microscope handling characteristics.
2) To perform statistical analysis to measure the reliability of metrics in grading surgeons' skill.

The related works in automated surgical skill assessment is furnished in the Section II. The materials and methods incorporated in the study for micro-neurosurgical skill analysis is detailed in Section III. Suturing, a mandatory surgical gesture in all neurosurgeries require careful handling of the tools and is elected as a good proxy to rate surgical skill. A quantitative discrimination of surgical skills of a neurosurgeon in suturing and the competence in microscope handling over years is presented in Section IV,V. The Results and Discussion and Conclusion are presented in Section VI and VII respectively. The proposed surgical skill assessment framework is an useful aid for educators to review, track and follow the performance of neurosurgery residents. To the best of our knowledge, this is the first such framework to explore micro-neurosurgical skill analysis over real patients.

## II. Related Works

The surgical competence is a blended outcome of knowledge, technical skills, decision making and team-handling skills of the surgeon. The competencies are commonly assessed either based on the observational approach through rating checklists or by patient outcome measures [5]. Even the eminent checklist based samples of observational category suffer criticisms of rater's subjectivity and evaluator's fatigue [5], [6]. The acute shortage in neurosurgeons also limits the checklist based assessments of residents. The outcome based evaluation metrics like morbidity, mortality, and re-admission highly rely on the type of the procedure and the physiological characteristics of the patient and does not effectively reflect competency [5], [7].

In recent years, the interest has shifted towards automated analysis of instrument and hand movement from the recorded videos as an effective alternative to access the psychomotor skills. [7], [18], [19] In this regard, several attempts have also been made to evaluate the surgeons' dexterity over the bench-top models, trainers, cadavers and simulators [8], [9], [10], [11], [12], [13], [14]. However the validation of the surgeon's performance over these models to the real skill used in surgery or to the patient outcomes have not been entrenched. In fact, an experimental study by Mills et al. [20] involving 10 surgeons with a median experience of 7.25 years highlights that there were no correlation between the surgeons' simulator performance and the ratings of their intraoperative videos by experts based on the Global Evaluative Assessment of Robotic Skills (GEARS) scale. This mandates the development of techniques to assess surgeon's competency in operating real patients. In this respect, Speidel et al. [15] analysed the motion behavior of a surgeon over Suturing, Knot-tying segments in a recorded video of Minimally Invasive surgery using the Conditional Density Propagation over time algorithm. In 2021, Goodman et al. [16] performed handpose analysis on Cutting, Tying, Suturing segments of open surgical procedures from Annotated Videos of Open Surgery (AVOS) Dataset harnessed from Youtube and highlighted the distinct surgical signatures of trainee and experienced surgeons. In the same year, Lavanchy et al. [17] developed a machine learning algorithm to analyse the clip application segment at the end of the hepatocystic dissection in laparoscopic cholecystectomy videos to automatically distinguish good versus poor surgical skill. Some of the relevant works on Surgical Skill Assessment using Surgeons' Motion Quality are presented in the Table I.

**TABLE I:** Related Works

**Author, Year**	**Procedure under Study**	**Sample Size**	**Parameters of Interest**	**Data Analysis Technique**
**In Trainer or Simulator**				
Suzuki, Takahisa, et al. 2015 [8]	Suturing	6 surgeons and 11 novices	Time and the Locus tracing	Linear Discriminant Analysis
Islam, Gazi, et al. 2016 [9]	Peg transfer, Intracorporeal suture, shape cutting	52 medical students and surgical residents	Hand and Tool motion features	Multivariable linear regression
Sharon et al. 2017 [10]	Teleoperated and open needle-driving with the da Vinci Research Kit	6 surgeons, 10 novices	Orientation metrics	Analysis of Variance
Wang et al. 2018 [11]	Suturing, Needle-passing, Knot-Tying	8 surgeons	Multivariate Time Series of Motion Kinematics	Deep Convolutional Neural Network
Fard, Mahtab J, et al. 2018 [12]	Suturing, Knot-tying	8 surgeons	Tool Motion Features	k'nearest neighbor, Logistic Regression, Support Vector Machine
Davids, Joseph, et al. 2021 [13]	Arachnoid Dissection Procedure	1 expert, 6 intermediate, 12 novice surgeons	Tool motion and Tool Handling metrics	Discriminant Analysis Classifier
Singh, Simar, et al. 2021 [14]	Cannulation in a Simulated ArterioVenous fistula	52 participants comprising of nurses, nurse practitioners, and dialysis technicians	Motion Smoothness Metrics	Linear Regression
**In Real Patients**				
Speidel, Stefanie, et al. 2006 [15]	Suturing, Knot-tying	1 surgeon	Tool Tracking	Conditional Density Propagation over time
Goodman, Emmett D., et al. 2021 [16]	Cutting, Tying, Suturing in open surgical procedures	14 operators including medical students, residents, surgeons	Hand Tracking and pose analysis	Simple, Online, Real-time Tracking Algorithm
Lavanchy, Joël L., et al. 2021 [17]	Segments of Clip Application	949 clip applications from the 242 laparoscopic cholecystectomy videos	Tool Motion Features	Linear Regression

## III. Materials and Methods

### A. Dataset

The dataset comprises of video recordings of a neurosurgeon performing variety of neurosurgeries like removal of gliomas, colloidal cyst and craniopharyngioma over real patients ranging from the year 2011 to 2017 in the Department of Neurosurgery, National Institute of Mental Health and Sciences (NIMHANS), India. All surgeries were carried out with the aid of Leica OH5 or Leica OH6 neurosurgery microscopes. And the video recordings of the surgery were acquired at the rate of 25 frames per second with the frame resolution of 640 × 480. The authors have taken approval from the NIMHANS ethics committee on $25/08/2022$ with the protocol No. $NIMHANS/37th IEC (BS \& NS DIV.)/2022$ and have permitted the usage of video recordings in this study. Suturing segments from 10 different surgeries are selected to study the tool handling characteristics of the surgeon over years. And the first 50 minute recordings of eight different neurosurgeries are selected to analyse the changes in pattern of microscope adjustments over the years of practice.

### B. Methods

An automated framework to segment microsurgical instruments and to characterize operating patterns using the instruments is presented in the Section IV. Mask RCNN coupled with efficient post-processing were employed to precisely segment five instruments namely Suction, Bipolar Forceps, Needle Holder, Straight Microscissors and Needle Holder from the neurosurgery videos. The position of the tool-tip and the orientation information of the tools employed in each frames were extracted using series of morphological and mathematical operations. Then the tool usage features to systematically analyse and grade surgical techniques is introduced. The operating microscope is an indispensable component of any neurosurgery. An object detection model to indirectly infer the microscope adjustments and the metrics to highlight the handling patterns are described in detail in the Section VI.

## IV. Microsurgical Tool Characterization

The operating maneuvers of a neurosurgeon are accredited as a combination of intuitive (subconscious) to analytical (conscious) actions [21]. The effective methodology in grading the level of technical expertise in neurosurgery is to analyse the movement of micro-surgical tools. Accurate segmentation and localization of micro-surgical tools forms the fundamental facet to construct an objective tool to evaluate the psychomotor skills. In this regard, the microsurgical tool segmentation dataset was built with a total of 4755 frames of resolution of 640 × 480 pixels extracted at 12 frames per second (FPS) from the neurosurgical videos. The frames are then annotated for the presence of 5 different instruments namely Suction, Bipolar Forceps, Needle Holder, Straight Microscissors and Needle Holder using the Labelme software [22].

### A. Tool Segmentation and Postprocessing

Instance segmentation of the surgical tools is the key to skill analysis. The Mask-RCNN is employed in the proposed model for instance segmentation of surgical tools from the neurosurgical videos. The Mask RCNN model is built on top of the Faster RCNN with the inclusion of small fully convolutional Neural network (FCN) applied to each region-of-interest (RoI) and predict the object mask for each instance in parallel to the existing branch for bounding box recognition [23]. The model is initialized with the pretrained weights of COCO dataset and later fine-tuned by training the network for 20 epochs with batch size of two to segment five surgical instruments namely Bipolar Forceps, Suction, Straight Micro Scissor, Straight Needle Holder and Dural Tooth Forceps.

**Fig. 1.**
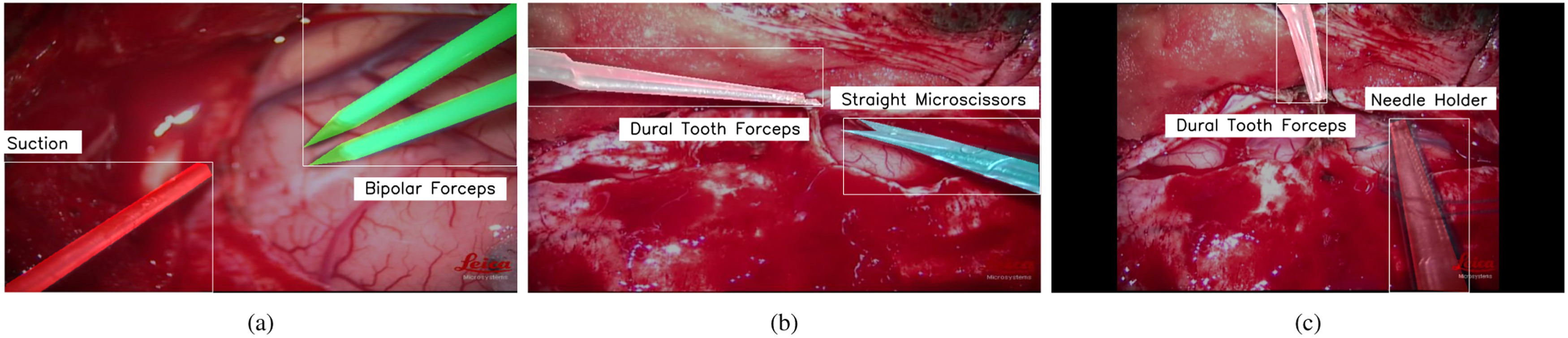
Tool segmentation results showing (a) Bipolar Forceps and Suction, (b) Dural Tooth Forceps and Straight Microscissors, (c) Dural Tooth Forceps and Needle Holder.

**Fig. 2.**
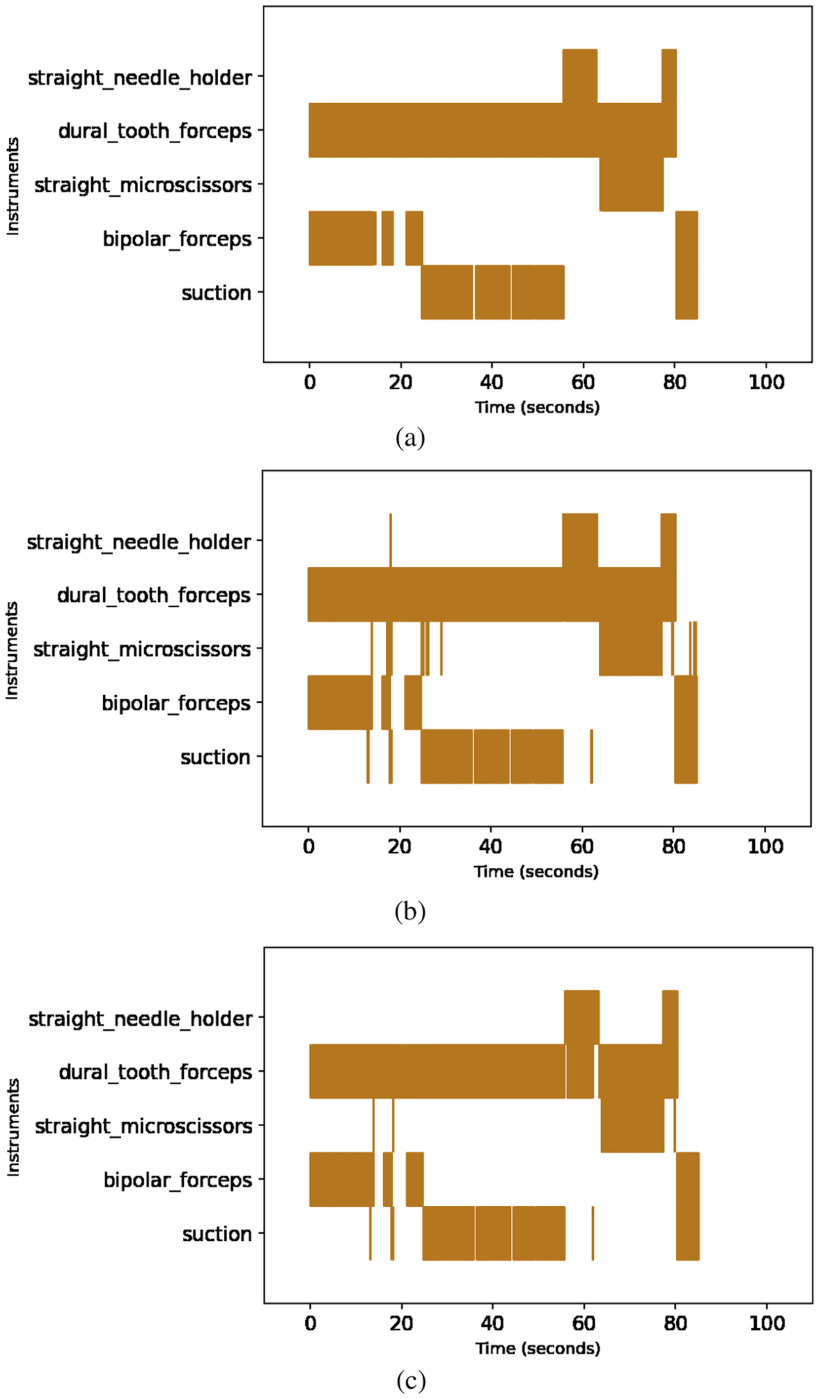
Tool Detection results in Neurosurgical Videos showing (a) Ground Truth On-Off Plot, (b) On-Off Plot before Post-Processing, (c) On-Off Plot after Post-Processing.

Micro-surgical tools have similar form factor and shape resulting in higher chances of misclassification. The false positive outputs are discarded by thresholding the confidence scores. Incorrect detections and mislabelled instances which are inherent consequences with the usage of frame based segmentation models over videos were handled efficiently with a robust and efficient post-processing (REPP) technique conceived by Sabater et al. [24]. The model was able to classify and segment five surgical tools from the recorded neurosurgical videos in real patients with a mean average precision of 0.967 at IoU of 0.5 despite occlusions, variations in the tool position, changes in the orientation as shown in the Fig. 1. The post-processing techniques augmented the detection results over the test videos as depicted in the Fig. 2. The methods and results of neurosurgical tool segmentation are detailed in our previous work [25].

### B. Tool Tip Detection and Angle Estimation

**Fig. 3.**
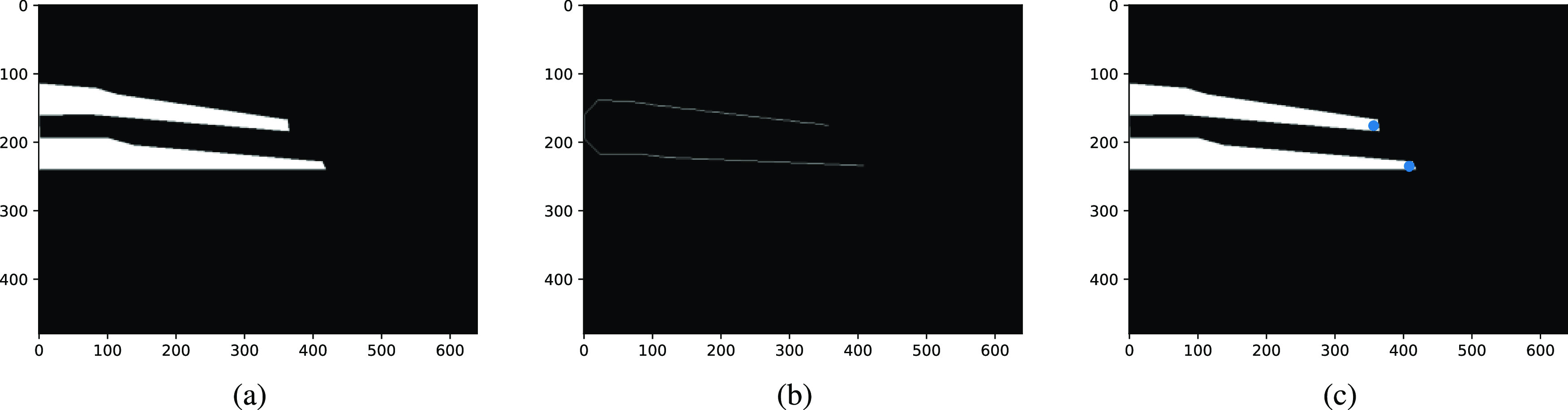
Tool Tip Detection showing (a) mask instance predicted by Mask RCNN, (b) medial axis skeleton of the mask instance, (c) tool tip overlayed over the mask.

Each predicted mask instances of surgical instruments are binarized by thresholding. The binary image of the surgical instrument is then subjected to medial axis skeletonization to extract the skeleton pixels through which no minimal path from any inner point to the shape boundary exists [26]. The tip of the surgical tool is extracted by examining the 8-connected neighbourhood of the skeleton pixels and the process is depicted in the Fig. 3.

**Fig. 4.**
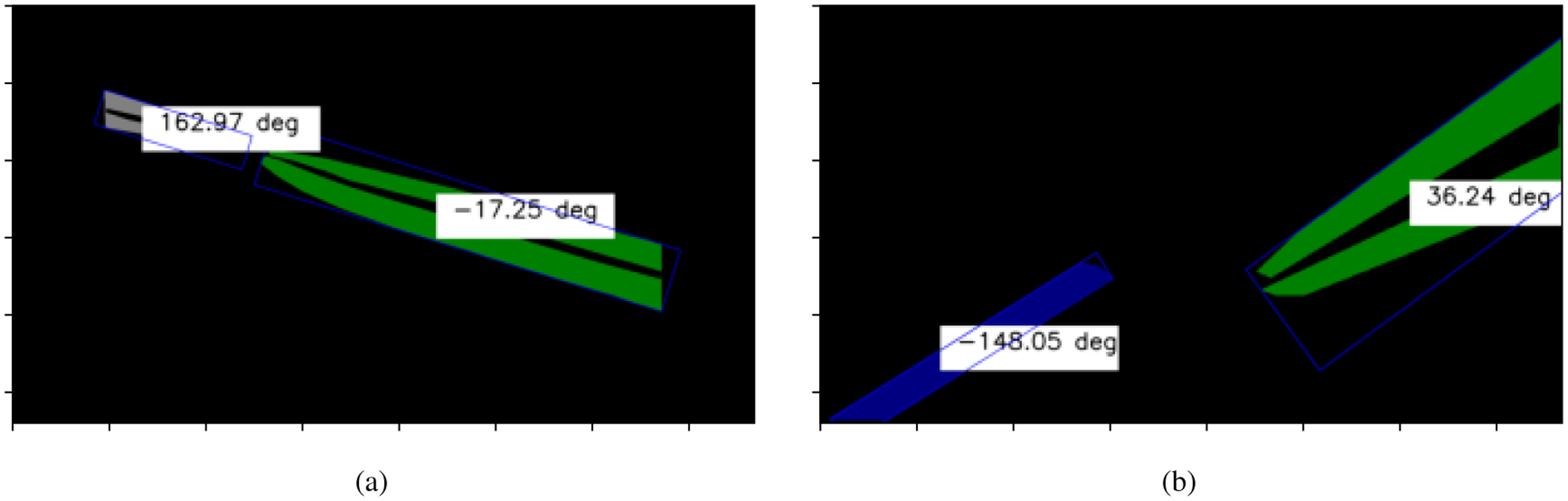
Surgical Instrument Orientation computed from the mask instances with the estimated angle in the text boxes.

For angle estimation, a minimum area rotated rectangle is determined for each instances of surgical instruments as shown in the Fig. 4. The orientation of the instruments in the surgical scene is computed as the angle made by long axis of the computed rectangle and the positive x-axis with vertex at the endpoint of long axis closer to the centre of the image.

### C. Tool Usage Feature Extraction

**Fig. 5.**
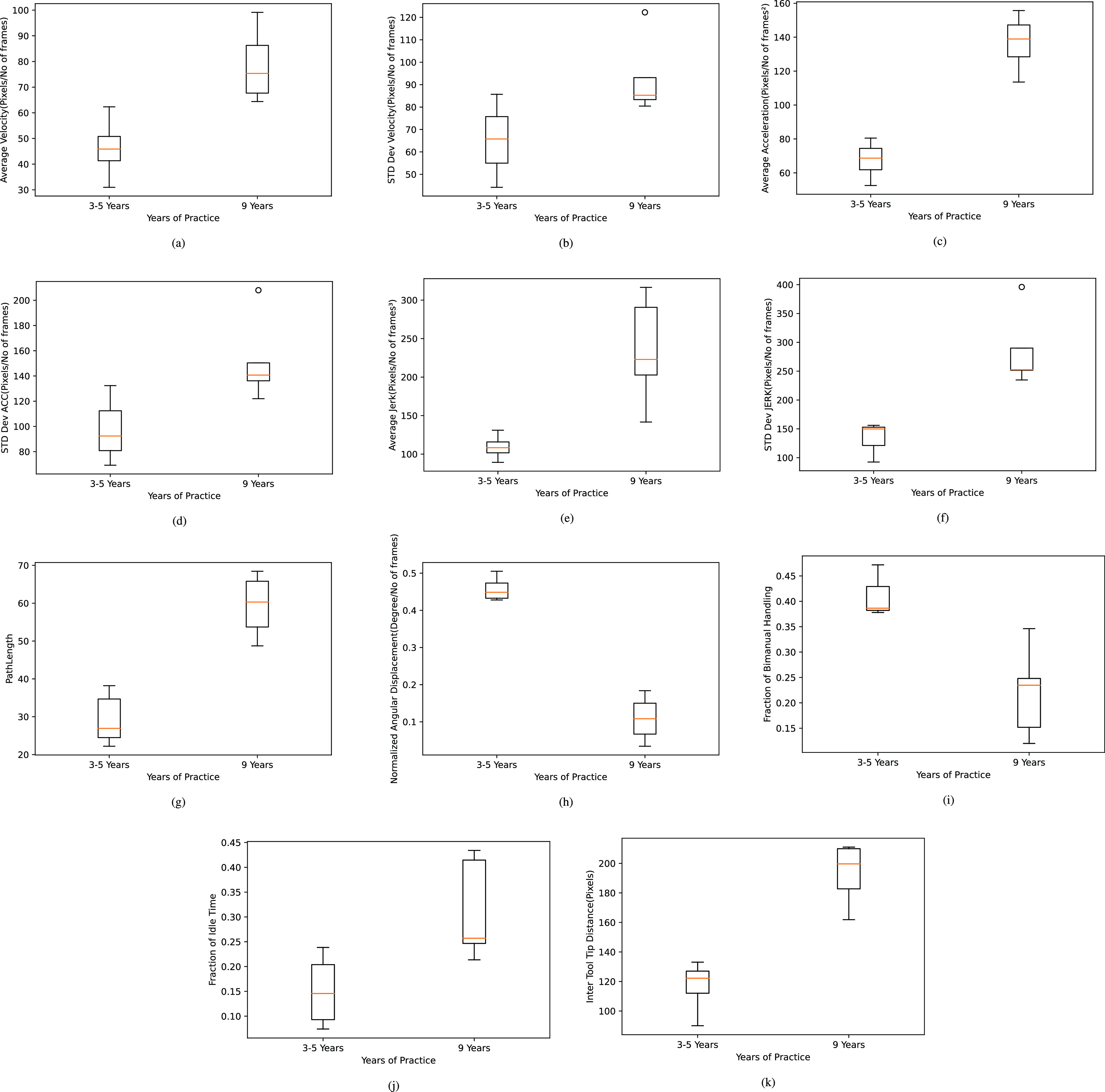
Box-plot comparison of tool based metrics as a function of years of practice. (a) Average Velocity. (b) Standard Deviation of Velocity. (c) Average Acceleration. (d) Standard Deviation of Acceleration. (e) Average Jerk. (f) Standard Deviation of Jerk. (g) Path Length. (h) Normalized Angular Displacement. (i) Fraction of Bimanual Handling. (j) Fraction of Idle Time. (k) Inter Tool-Tip Distance.

The Suturing segments from 10 different neurosurgeries performed by a neurosurgeon from the year 2011 to 2017 are analysed to decipher the parameters that could elicit distinctive and notable improvements in the surgical skill over time. Five video segments from the year 2011 to 2013 and five segments from the year 2017 are analysed to objectively compare the improvement in dexterity with the transformation of the surgeon into a veteran. All instruments in the surgery were hand-held and no robotic assistance was utilized to reduce resting tremor. From each video recording, the frames are extracted at the rate of 12 per second. Then the extracted frame sequences are subjected to Mask RCNN model followed by post-processing to segment the desired surgical tools. The 2-Dimensional coordinates of the segmented tool's tip and the orientation of the tools are then computed as described in the Section IV-B. Then the tool motion characteristics and the tool handling efficiency are assessed through the following metrics: Mean Velocity, Standard Deviation of Velocity, Mean Acceleration, Standard Deviation of Acceleration, Mean Jerk, Standard Deviation of Jerk, Normalized Angular Velocity, Bimanual Handling of tools, Idle Time and Inter Tool Tip Distance. These metrics are described and mathematically expressed in Table II. Fig. 5 presents these characteristic parameters over the year of practice.

**TABLE II:** Metrics to Characterize Tool Movements

**Metric**	**Description**	**Equation**
Velocity Metrics	Measure of the rate of change of Tool tip position $(x_{i},y_{i})$. Average Velocity $\bar{v}$ and Standard Deviation of Velocity $\sigma _{v}$ derived from computed velocity, $v_{i}$ are used as metrics	$v_{i}=\sqrt{(\frac{x(i+1)-x(i)}{dt})^{2}+(\frac{y(i+1)-y(i)}{dt})^{2}}$ $\bar{v}=\frac{1}{N}\sum _{i=1}^{N} v_{i}$ $\sigma _{v}=\sqrt{\frac{\sum (v_{i}- \bar{v})^{2}}{N}}$
Acceleration Metrics	Measure of the rate of change of Tool tip velocity. Average Acceleration $\bar{a}$ and Standard Deviation of Acceleration $\sigma _{a}$ derived from computed Acceleration, $a_{i}$ are used as metrics	$a_{i}=\frac{v(i+1)-v(i)}{dt}$ $\bar{a} =\frac{1}{N}\sum _{i=1}^{N} a_{i}$ $\sigma _{a}=\sqrt{\frac{\sum (a_{i}- \bar{a})^{2}}{N}}$
Jerk Metrics	Measure of the rate of change of Tool tip Acceleration. Average Jerk $\bar{j}$ and Standard Deviation of jerk $\sigma _{j}$ derived from computed Jerk, $j_{i}$ values are used as metrics	$j_{i}=\frac{a(i+1)-a(i)}{dt}$ $\bar{j} =\frac{1}{N}\sum _{i=1}^{N} j_{i}$ $\sigma _{j}=\sqrt{\frac{\sum (j_{i}- \bar{j})^{2}}{N}}$
Path Length	Measure of total distance travelled by the tool	$PL=\sum _{i=1}^{N}\sqrt{(x(i+1)-x(i))^{2}+(y(i+1)-y(i))^{2}}$
Normalized Angular Velocity	The accumulation of change in tool orientation, $\theta _{i}$ normalized by the Path Length	$A =\frac{1}{PL}\sum _{i=1}^{N} \frac{\theta (i+1)-\theta (i)}{dt}$
Fraction of Idle Time	Measure of fraction of frames where no tools are present	$IT = \frac{Number\; of\; frames\; with\; no\; tools}{Total\; number\; of\; frames}$
Fraction of Bimanual Handling	Measure of fraction of frames where surgeons uses two tools bi-manually	$BH = \frac{Number\; of\; frames\; with\; bimanual\; tools}{Total\; number\; of\; frames}$
Inter Tool-Tip Distance	Measure of the Euclidean distance between Tool tip positions when more than one tool is present	$TTD =\frac{1}{N}\sum _{i=1}^{N}\sqrt{(x_{1}(i)-x_{2}(i))^{2}+(y_{1}(i)-y_{2}(i))^{2}}$

## V. Microscope Movement Characteristics

The advent of microscope into the operating room by Nylen in 1921 revolutionized the surgical practices [27]. With further refinements, the adjustable magnification, brighter illumination and the lucid visualization of the surgical field offered by the operating microscope have made it indispensable in the field of modern neurosurgery [28]. A thorough knowledge on practical operations enables improved handling of the microscope and the surgeon become progressively proficient with its use. The correct usage and proper handling is critically important in the success of complex surgical interventions [29]. And we hypothesize that there is a difference in microscope adjustment patterns with expertise in neurosurgeons and therefore we intend to introduce metrics to highlight the variations.

In the operating microscopes, when the focusing aid is active, the focusing lasers are triggered. Hence, any microscope adjustments is indirectly inferred from the presence of the two red colored laser spots on the image. Detecting and tracking of the laser dots in the recorded videos helps to analyze the adjustment patterns.

### A. Laser Dots Detection

**Fig. 6.**
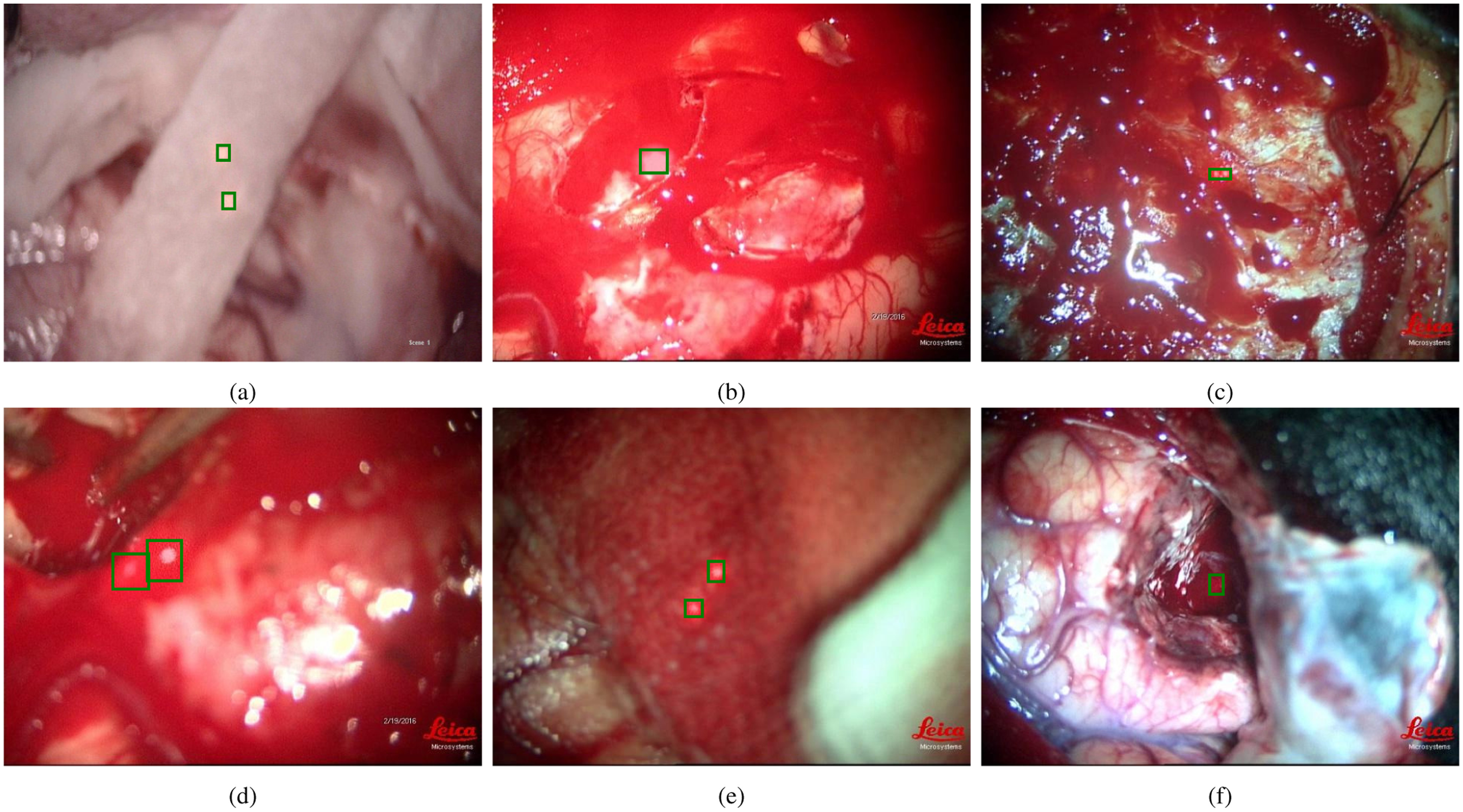
Yolov5 Laser Dots Detection Results overlaid over the images.

**Fig. 7.**
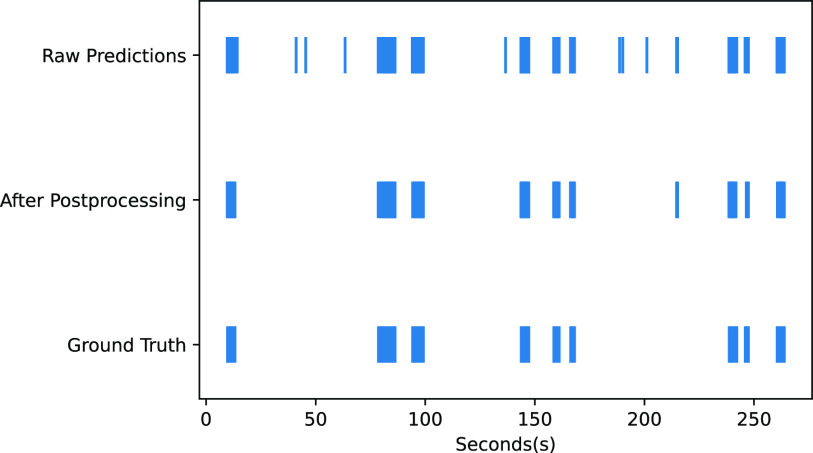
Laser Dots on-off Plot.

Yolov5 which belongs the family of single-stage deep learning framework for object detection was employed to detect the laser spots. Yolov5 employs CSPDarknet53 as backbone for feature extraction which feeds into a path aggregation network (PANet) for feature fusion and followed by the YOLO head to generate predictions [30]. Yolov5 model was intialized with the pre-trained weights extracted from MS COCO dataset, and the detection head was modified to have 2 classes to differentiate the background and the laser spots. The model was then fine-tuned with the annotated training dataset for the detection of red colored laser dots. Post-processing is inevitable in small target detection and REPP proposed by Sabater et al. [24] was employed to improve detection. Fig. 6 shows few samples of images with the predicted laser dots that are overlaid. Fig. 7 shows the on-off timings of laser dots as predicted by the model over 275 seconds duration of neurosurgical video. The on-off plot presents the laser dot predictions over the ground truth. The REPP based post processing clearly shows the improvement over the raw predictions and it closely matches with the ground truth results.

### B. Microscope Adjustment Features

Neurosurgeons are required to be very adept in handling microscope as almost all surgeries of brain and spine inclusive of all complex delicate procedures are carried out with the aid of microscope. With the aid of operating microscopes, there is minimal chance of disturbance to the neighbouring regions of abnormality even in intricate neuro-procedures thereby resulting in improved patient outcomes.

**Fig. 8.**
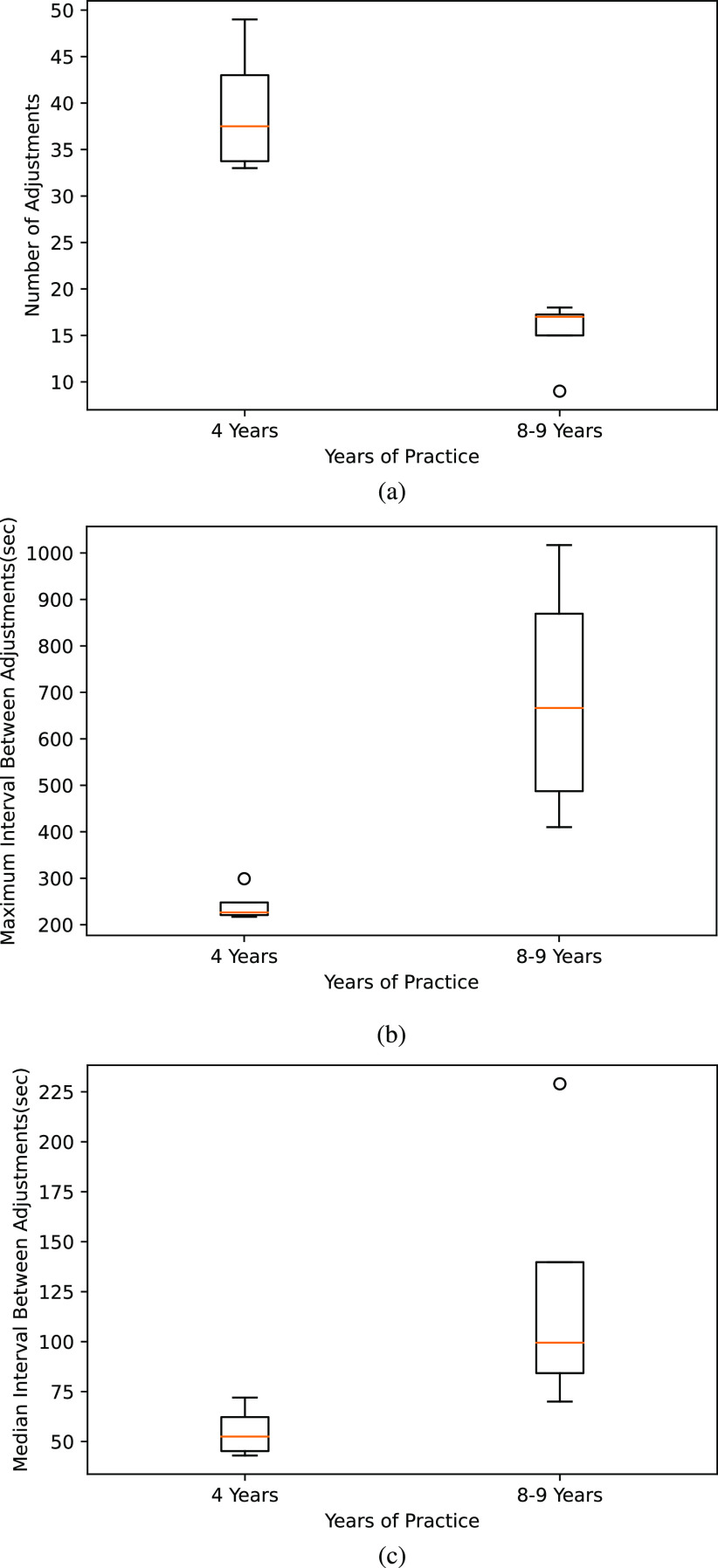
Box-plot comparison of Microscope Adjustment pattern based metrics as a function of years of practice. (a) Number of Microscope Adjustments. (b) Maximum Interval Between Adjustments. (c) Median Interval Between Adjustments.

In micro-neurosurgery, the perceived image space under the magnification offered by microscope is different from the actual. The major challenge to the neurosurgeon is to get trained to assimilate tactile feedback from the instruments and the visual information from the microscope and to automatically compensate for the perceptual mismatch from experience [31]. Master neurosurgeons typically handle the microscope effectively throughout in a subconscious mode whereas the less-skilled surgeons tend to use the microscope during relatively less challenging situations and avoid during inevitable situations like hemorrhage or when unusual and complex problems occur [31]. Hence measurable parameters are introduced in this paper to assess the competency of a surgeon in handling operating microscope. However the neurosurgical phase specific challenges also play an important role in microscope manipulation pattern. In this regard, microscope adjustment pattern is investigated in the first 50 minutes of the surgery when the size of the tumor or any other complexity in the surgical procedure does not have greater influence. The first 50 minute video recordings from four neurosurgeries in the year 2012 and four segments from the year 2016–2017 performed by the same neurosurgeon are analysed to inspect variations in the usage pattern as the surgeon becomes more proficient in handling operating microscope and the metrics are presented in the Fig. 8. Along the years of practice, the number of microscope adjustments is shown to reduce, whereas the maximum and median interval between adjustments is observed to increase.

## VI. Results and Discussion

Efficient handling of tools and operating microscope are salient traits of an experienced neurosurgeon. This study aims to establish the useful metrics that assesses the improvements in micro-neurosurgical skills of a neurosurgeon over the years of practice. Mann-Whitney U test is employed to study the difference in the metrics between the two range of years as it does not rely on distributional assumptions.

Multiple tools like Needle Holder, Straight Microscissors, Dural Tooth Forceps and Suction have a role during suturing. This is very pertinent to examine the optimal multi-tool handling, tool switching, task planning and sequencing ability of a surgeon and hence analysis of suturing segment is opted in this study. The tool handling metrics are evaluated and compared between the suturing segments of ten neurosurgery videos of a neurosurgeon recorded between the years 2011 to 2013, and 2017. The initial step for computing tool-based metrics involves detecting and localizing the different micro-neurosurgical tools using Mask-RCNN. Then the tip of the surgical tool is tracked to compute the tool-handling metrics. The parameters are sensitive enough to discriminate various levels of expertise of a neurosurgeon as summarized in the Table III. The lower velocity, acceleration and jerk related metrics with experience is the consequence of good motor control and the ability to make smoother and economical movements with maximal efficiency. With experience, the surgeon is also able to handle the tool optimally making precise movements with shorter trajectories thereby resulting in smaller path lengths (median 26.94 vs 60.31 & p = 0.0061). Normalized Angular displacement is also observed to be significantly higher with a proficient surgeon during suturing (median 0.45 vs 0.11 & p = 0.0152). In general, an expert demonstrates familiarity through planned course of actions with uninterrupted task flow and utilizes both hands with optimal interaction [32], [33]. Hence, a beginner exploiting a longer idle time for execution of the task is attributed to the deficit in knowledge of surgical techniques and prolonged duration for planning and decision making (median 0.15 vs 0.26 & p = 0.0187). And a good bi-manual dexterity (median 0.39 vs 0.23 & p = 0.0184) with meticulous spatio-temporal coordination of both the hands indicated by lower inter tool-tip distance (median 122.21 vs 199.67 & p = 0.0158) is perceived with experience.

**TABLE III:** Summary of the Tool Handling and Microscope Handling Metrics of a Neurosurgeon Over Years of Practice in Relation to the Assessment of Micro-Neurosurgical Skills

**Metric**	**Median Value**		**p-value**
		**Expert**		**Novice**	
**Tool Handling Based Metrics**			
Average Velocity	45.89	68.77	0.0331
Standard Deviation of Velocity	65.76	85.27	0.1165
Average Acceleration	68.70	133.44	0.0187
Standard Deviation of Acceleration	92.41	140.7	0.0368
Average Jerk	108.30	222.93	0.0100
Standard Deviation of Jerk	149.81	252.50	0.0184
Path Length	26.94	60.31	0.0061
Normalized Angular Displacement	0.45	0.11	0.0152
Fraction of Idle Time	0.15	0.26	0.0187
Fraction of Bimanual Handling	0.39	0.23	0.0184
Average Inter Tool Tip Distance	122.21	199.67	0.0152
**Microscope Handling Based Metrics**			
Number of Microscope Adjustments	38	17	0.0147
Maximum Interval Between Microscope Adjustments	227	667	0.0152
Median Interval Between Microscope Adjustments	53	100	0.0152

The first fifty minute duration of eight recorded neurosurgeries performed by a neurosurgeon over the years 2011–2012 and 2016–2017 are analysed to study the operating microscope adjustment patterns and summarized in the Table 3. The instant of microscope adjustments is indirectly inferred from the red laser dots that overlay on the images when the focusing aid is active. Yolov5 is employed in this study to detect the laser dots. It is observed that there is a pronounced increase in the frequency of microscope adjustments (median 38 vs 17 & p = 0.0147) and drop in the maximum interval (median 227 vs 667 & p = 0.0152) and median interval (median 53 vs 100 & p = 0.0152) between adjustments with experience of a neurosurgeon. Over years of experience, the neurosurgeon gets more acquainted to microscope and is not reluctant to make adjustments when necessary.

## VII. Conclusion

This study detailed a framework for automated objective assessment of micro-neurosurgical skills and this is the first reported study on assessment of real-life neurosurgery rather than a bench-top task. The proposed video based methodology is designed to assist residents to measure and compare the tool handling and microscope handling characteristics with explainable standardized metrics. And to the best of our knowledge, this is the first time operating microscope adjustment features have been reported in micro-surgical skill analysis. The proposed technique has potential to offer real-time feedback and shows promise as a reliable and valid method to track performance over time and to accomplish meaningful comparison. This pilot study has proven that it is feasible to completely automate the hassle process of surgical skill assessment in residents and is definitely a valuable contribution in the direction of automated surgical skill assessment. Our ongoing research and the future direction in this field is to establish a structured grading system for surgeons to assess micro-neurosurgical proficiency's in an uniform scale.
